# Employment of a noninvasive magnetic method for evaluation of gastrointestinal transit in rats

**DOI:** 10.1186/1754-1611-6-6

**Published:** 2012-05-15

**Authors:** Caio C Quini, Madileine F Américo, Luciana A Corá, Marcos FF Calabresi, Matheus Alvarez, Ricardo B Oliveira, Jose Ricardo A Miranda

**Affiliations:** 1 Instituto de Biociências de Botucatu, IBB – Universidade Estadual Paulista – UNESP, Distrito de Rubião Jr s/n, Botucatu, São Paulo, CEP: 18600-000, Brazil; 2 Instituto de Ciências Biológicas e da Saúde, UFMT – Universidade Federal de Mato Grosso, Barra do Garças, Mato Grosso, Brazil; 3 Pró-reitoria de Pesquisa e Pós-Graduação, UNCISAL – Universidade Estadual de Ciências da Saúde de Alagoas, Maceió, Alagoas, Brazil; 4 Faculdade de Medicina de Ribeirão Preto, USP – Universidade de São Paulo, Ribeirão Preto, São Paulo, Brazil

**Keywords:** Biomagnetism, Gastric emptying, Gastrointestinal markers, Liquid meal

## Abstract

AC Biosusceptometry (ACB) was previously employed towards recording gastrointestinal motility. Our data show a reliable and successful evaluation of gastrointestinal transit of liquid and solid meals in rats, considering the methods scarcity and number of experiments needed to endorsement of drugs and medicinal plants. ACB permits real time and simultaneous experiments using the same animal, preserving the physiological conditions employing both meals with simplicity and accuracy.

## Background

Gastrointestinal (GI) motor activity consist of an intricate group of functions that are essential for life [1], and disorders of GI transit and/or contractility are common [2]. Gastric emptying is complex and reflects a variety of functions which include accommodation and coordinated relationship between the proximal/distal stomach and antropyloroduodenal contractility [3]. The orocacaecal transit time is also multifaceted and depends on gastric emptying, small intestine motility and ileocaecal junction activity [4].

A number of techniques have been employed to evaluate GI motility and transit, but few techniques are able to evaluate more than one GI motility parameter simultaneously [3].

GI transit can be quantified in rats, by measuring the movement of charcoal, dye, radiopaque markers or other non-absorbable materials [5,6]. This procedure requires the sacrifice of a large number of animals in order to determine the propulsion of such markers within the gut at predetermined time intervals and usually measurements of gastric emptying and small bowel transit involves separate groups of animals [5].

Scintigraphy is the gold standard method for gastric emptying in humans [7,8]. Such investigations are performed employing radiolabeled meals; however the costs, radiation exposure, licensing for handling radioactive materials and approval by appropriate institutional committee as well as limited temporal and spatial resolution are some of the drawbacks of this technique, especially when considering animal studies [9,10]. Breath hydrogen test is a noninvasive technique that was utilized in some animal studies despite of serious pitfalls in data interpretation [2,11].

Alternating Current Biosusceptometry (ACB) is an inexpensive, radiation-free and noninvasive method that was previously employed as a reliable technique to record GI transit and contractility in humans, dogs and rats. ACB data showed accuracy and close agreement with standard techniques in humans and dogs [12-15]. Recently, ACB was validated for monitoring gastric contractility in rats using strain-gauges transducers as the gold standard method [16]. These studies were performed with solid or semisolid meal because there was no liquid magnetic marker that could be used by that time. Despite the importance of evaluating the GI transit when medicinal plants and drugs are tested, the ACB has not been employed for this purpose in rats yet.

The aim of this study was to monitor in real time GI transit of liquid and solid magnetic meals by using ACB technique in order to establish this biomagnetic method as a reliable technique for multiple records of the GI motility in the same rat.

## Results

### Technique

A single ACB sensor works as a double magnetic flux transformer without any nucleus and has two coaxial pairs of coils separated by a baseline of 15 cm [13,17]. Each pair is composed of an excitation coil (outer) and a detection coil (inner) in a first-order gradiometric configuration that provides good signal-to-noise ratio. One pair works as the reference and the other as the detector probe. Basically, the excitation coil works with a frequency of 10 kHz generated by lock-in amplifiers and a current of 88 mA that produces a magnetic field of 20 G (rms) and induces equal magnetic flux in the detection coils. Hence, when the magnetic sample is nearest to the sensor an imbalance in the voltage occurs, due to the changes in the differential flux between the detection coils. The ACB sensor can locate the magnetic material through magnetic flux variation between these coils. The signal intensity detected by the sensors depends on the surface area of the detection coil, number of turns, rate of change of the magnetic flux (i.e. applied field and frequency), the amount of magnetic material as well as the distance between the sensor and the magnetic sample [16]. For this study, the ACB sensor was developed with excitation coils (ϕ= 3.5 cm; 200 turns of 26-AWG wire) and detection coils (ϕ = 2.9 cm; 500 turns of 32-AWG wire) to improve spatial resolution and sensitivity for laboratory animals.

Ferrite powder (Imag, Brazil) utilized in solid meals (Fe_2_MnO_4_ – microparticles 50 ≤ ϕ ≤ 100 μm) remained completely inert in all pH solutions and cannot be absorbed by GI tract due to its inter-molecular ligations and size. The ferrofluid (fluidMAG-Chitosan, Chemicell, Germany) employed in liquid meals (nanoparticles with diameter of 200 nm) consists of iron oxide magnetic particles that were coated with chitosan by spray-drying method. Based on earlier studies, it is reasonable to assume that these particles are not absorbed by mucosa in the GI superior tract [18]. In both situations, signals have been obtained from magnetic material that was dispersed in the GI lumen.

### Animals

Twenty-four individually housed male Wistar rats (weighting 300–350 g) were used in the study. All procedures were performed in accordance with the Guide of the Care and Use of Laboratory Animals (Brazilian College of Animal Experimentation) and were approved by the local Animal Ethics Committee. Animals were fasted 20 h before experiments, with free access to water. For comparison tests, six groups with three animals each were killed at pre-determined time intervals (10 min) after ingestion of solid meal similarly as it is performed in activated charcoal test. In vivo tests, the studies (liquid and solid meals) were performed in the same animal in a randomized order and were separated by an interval of 1 wk.

### Recording of gastrointestinal transit

**Figure 1:**
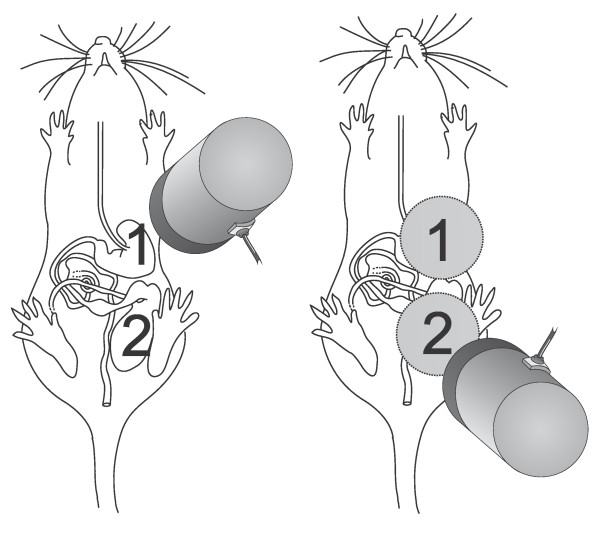
**Diagram showing the positioning of the ACB single-sensor (open circles) on the rat abdominal surface.** The animals were handled gently by the neck and the sensor was positioned on their gastric (1) and cecal (2) projection after ingestion of either solid or liquid magnetic meals.

Magnetic monitoring was achieved by measuring the intensity values recorded by the single-sensor ACB (Br4-Science®, Brazil) placed on abdominal surface. The animals were handled gently by the neck and the sensor was positioned on their gastric and cecum projection after ingestion of either solid or liquid magnetic meals (Figure 1).

Liquid meal: Ferrofluid (1.0 ml – 50 mg/mL) diluted in 1.5 ml of distilled water was administered by gavage to rats and five minutes later the abdominal surface was scanned by ACB sensor. The point of maximum magnetic signal intensity was identified as corresponding to the stomach and the magnetic value was registered. After that, the ACB sensor was placed in the cecum projection (chosen on the basis of anatomical references) and the magnetic signal intensity was also recorded. Subsequent measurements were made in awake rats at these two points at regular 10-min intervals for at least 7 hours [19].

Solid meal: Pellet (2 g) made of powder ferrite (0.5 g) and laboratory chow (1.5 g) was quickly ingested by the animals, 10 min before starting the experiments. The abdominal surface was scanned by ACB sensor following the same protocol described above for liquid meal.

### Data analysis

All raw signals were analyzed in MatLab (Mathworks, Inc., USA) by visual inspection and the statistical moment was calculated. The statistical moment was obtained through the temporal average pondered by magnetic intensity curves, normalized by area under curve [20]. By using this approach, the following parameters were quantified: Mean Gastric Emptying Time (MGET) was defined as the time t (min) when a mean amount of magnetic meal was emptied of the stomach and it was calculated by the area under emptying curve; Mean Cecum Arrival Time (MCAT) was defined as the time t (min) when occurred a increase in mean amount of magnetic meal that arrived in cecum and it was calculated by the area between cecum arrival curve until maximal cumulative values; Mean Small Intestinal Transit Time (MSITT) was quantified as the difference between MCAT and MGET.

All the results are expressed as mean ± standard deviation (SD). Values of MGET, MCAT, and MSITT obtained after ingestion of liquid or solid meal were correlated. By using paired Student’s t-test statistically significant difference was considered at *p* < 0.05.

#### Comparison tests

A magnetic pellet (ferrite) was quickly ingested by animals (n = 18) and 10 minutes later the experiments starts. The animals have gastric magnetic intensity recorded and immediately after that, at pre-determined time intervals (10, 20, 30, 40, 50 and 60 min), they were killed. The small intestine was carefully removed and the distance traveled by ferrite (in analogy to activated charcoal) up to the last portion that contained at least a continuous 1 cm trace of this marker was determined. The results were expressed as intensity (mV) and distance (cm) traveled by ferrite.

**Figure 2:**
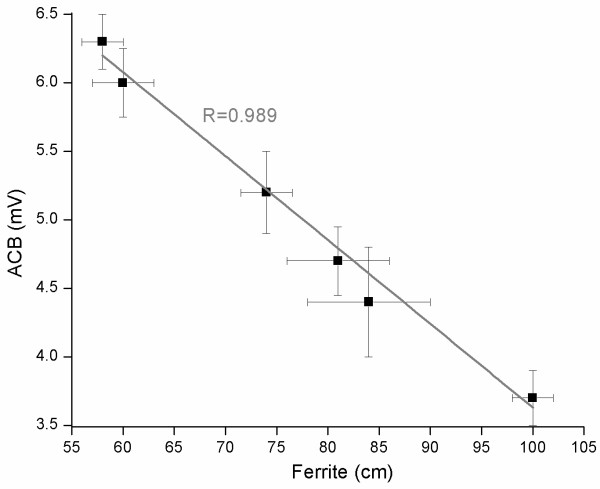
**Correlation between gastric magnetic intensity (mV) recorded by ACB and the distance traveled by magnetic tracer in small intestine (cm), before and after animals be killed, respectively.** Standard deviation was presented as error bar (vertical and horizontal for intensity and distance, respectively) for both measurements, assuming n = 3 for each point that corresponding to sequential measurements each 10 minutes. The gray line represents the linear correlation between methods used to evaluate GI transit.

Figure 2 showed that displacement of material in small intestine had high correlation (0.9) and linearity with decrease in gastric magnetic signal intensity, corroborating the ACB technique.

#### In vivo tests

The GI transit times parameters for liquid and solid meals are summarized in Table 1. As expected, gastric emptying time was markedly different between both meals in the same animal. MGET values quantified for liquid and solid meals were 99.58 ± 13.50 min and 140.52 ± 35.51 min, respectively. Statistically significant differences were obtained between the liquid and solid values of MGET (*p* < 0.04).

**Table 1:** MGET (mean gastric emptying time), MCAT (Mean cecum arrival time) and MSITT (mean small intestinal transit time) in minutes for liquid and solid meals calculated using statistical moments

	**Liquid meal**			**Solid meal**		
Rat	MGET	MCAT	MSITT	MGET	MCAT	MSITT
1	119	209	90	118	263	145
2	100	194	94	125	181	56
3	83	194	111	111	173	62
4	91	184	93	158	284	126
5	92	231	138	203	334	131
6	112	206	94	129	228	99
Mean	100	203	103	141*	244*	103
SD	14	16	19	35	62	37

Mean ± SD (min); * *P* < 0.04 vs. liquid meal.

MCAT values for liquid and solid meals were 202.86 ± 16.31 min and 243.74 ± 62.12 min, respectively. MCAT also presented a significant increase for solid meals (*p* < 0.04). The mean values of MSITT on both liquid and solid meals were 103.28 ± 18.73 min and 103.22 ± 37.26 min, respectively. There is no statistically significant difference between the liquid and solid values for MSITT.

**Figure 3:**
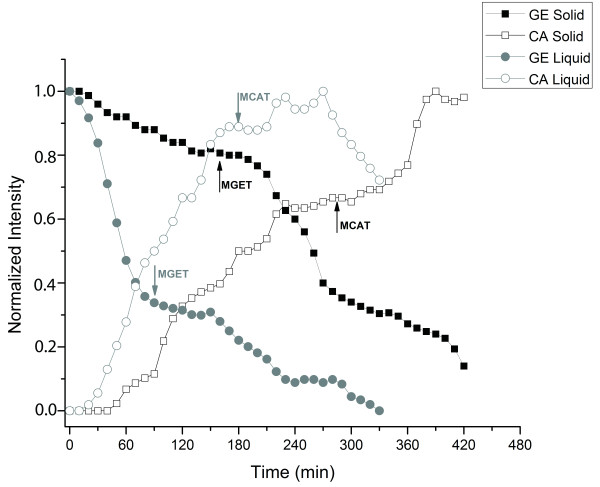
**Typical gastrointestinal transit profiles represented by the example obtained for rat number 4.** Gray symbols correspond to gastric emptying (closed circle) and colon arrival (open circle) for liquid meal. Black symbols correspond to gastric emptying (closed square) and colon arrival (open square) for solid meal. The arrows indicate the MGET (mean gastric emptying time), MCAT (Mean cecum arrival time) and MSITT (mean small intestinal transit time).

Gastric emptying (MGET), cecum arrival (MCAT) and small intestinal transit (MSITT) profiles obtained by ACB after ingestion of liquid (gray cicles) and solid meals (black squares) are illustrated in the Figure 3.

A variability profile was obtained using the same animal (n = 3) in three ACB recordings with one week of interval among them. The biological variability in these measurements was determined in around 6 % for MGET and 11 % for MCAT.

## Discussion

Our data show that ACB is useful for monitoring gastric emptying, cecum arrival and small intestinal transit time using both liquid and solid meals. The ACB is a flexible method combining reliability assessment of GI transit and does not require anesthesia or death for studies using laboratory animals.

It is important to emphasize that there are specific techniques for measurements of emptying, contractility, accommodation and sensation [21], however ACB is feasible to record more than one parameter simultaneously concerning GI motility. The comparison performed after solid meal ingestion using magnetic material instead of activated charcoal showed the relationship between gastric emptying and GI transit. The linear correlation between distance traveled by magnetic material on small intestine and decrease in gastric magnetic signal intensity was very strong (Figure 2), despite the variation observed in both approaches. ACB and displacement of material in GI tract have different principles, but our results suggested that gastric emptying of magnetic material corresponds linearly to its displacement in small intestine.

As previously demonstrated, gastric emptying profile can be obtained by ACB because when magnetic material moves into duodenum the intensity of magnetic signal decreases in a point representative of the stomach. Likewise, signal intensity increases when material arrives to the cecum and it is also possible to evaluate the orocaecal transit time [19].

In this study, ACB was employed for the first time to evaluate magnetic liquid meal since this was greatest challenge for this biomagnetic technique for several years. Hereafter, the nanotechnology provides an evaluation of liquid transit that can be compared with solid allowing to establish a complete profile for GI transit times.

The gastric emptying of noncaloric liquids meal has usually been exhibited as exponential pattern, while after solid meal the emptying have been characterized by a significant delay [22,23]. Typically, for gastric emptying, the average time (t_50_) is adopted and consists of the signal decay by half-intensity. This procedure has been severely criticized because is associated with a pure exponential model, which is not realistic for most of the processes of emptying [20]. Meanwhile, for the analysis of cecum arrival time, each method employs a measurement procedure, which is extremely variable and dependent on the analysis adopted. In order to quantify the gastric emptying and orocaecal transit time, we used the statistical moment that was previously utilized mainly in pharmaceutical approaches. The statistical moments provide accurate and less subjected information about the parameters analyzed because the mean values obtained representing the whole process. These values are obtained by analyzing the entire curve formed during the experimental procedure and also by the area under curve [20].

As expected, gastric emptying for liquid meal has a shorter time than solid; the arrival process of food to the cecum is not concurrent with the emptying, as occurs for solid meal. Even before of the complete gastric emptying, magnetic material have already been detected on caecum region. This profile can be explained by the time it takes for meal leaving the stomach and travel through the bowel of the animal. Still, it is important to note that the average time of arrival of meal in the cecum (MCAT) for liquid meal is considerably less than the same coefficient for solid meal (*p* = 0.04). As reported by others, our data showed that small intestinal transit time (MSITT) of both meals exhibited essentially the same transit rates [23].

Several physiological conditions can be responsible for an altered GI transit [24]. A slow orocaecal transit during pregnancy was extensively documented but poorly understood [25]. Several disorders can be associated with abnormal gastric emptying rate such as diabetes mellitus and with GI transit changes such as diarrhea and colitis [26,27]. Recently, medicinal plants have received more and more attention aiming its clinical application, despite concerns about their reliability and safety analysis [6]. The endorsement of drugs and medicinal plants requires several assessments, and using the traditional methods can lead to death of several animals in each of them. Our technique has potential to demonstrate both enhancement and inhibition of gastric emptying and orocaecal transit time and helps to understand better all these situations. ACB uses much smaller number of animals and had accuracy comproved by the displacement of material.

The potential weaknesses of the current study are the multiple handling of the animal that is known to increase plasma cortisol and catecholamines which potentially may confound stress effects with the chosen experimental stimuli [5]. However, the animals were trained for months by the same researcher being handled gently several times a day minimizing these unwanted effects. This approach ensures good care and minimizes the handling impact on experimental results [28].

Animals have been used as experimental models for centuries and their use has enabled researchers to make significant advances in many areas of human health and disease [29]. Nowadays, is essential to take into account ethical considerations carefully before starting an experimental design [30]. The experimental procedure described here allows multiple measurements of GI transit in the same animal with simplicity and accuracy. The improvement of ACB sensor for laboratory animals (rats) in association with new magnetic fluids can contribute for real-time evaluation of important parameters concerning the GI motility.

## Conclusions

Our magnetic technique allows the in vivo experiments, have a high correlation with standard technique for GI transit in rats and preserving the physiological conditions. Also, both liquid and solid magnetically marked meals may be used paralleling a normal diet and this protocol can be applied in drugs and medicinal plant tests without unnecessary animal death.

## Competing interests

The authors declare that they have no competing interests.

## Authors’ contributions

AMF, ORB and MJRA designed the research; QCC, AM and CMFF performed the research; QCC, AM and ORB contributed analytic tools; QCC, CMFF and MJRA analyzed the data; AMF and CLA wrote the paper. All authors have read and approved the final manuscript.

## References

[B1] Huizinga JD Lammers WJ Gut peristalsis is governed by a multitude of cooperating mechanisms Am J Physiol 2009 296 G1 G8 10.1152/ajpgi.90380.2008 18988693

[B2] Rao SS Camilleri M Hasler WL Evaluation of gastrointestinal transit in clinical practice: position paper of the American and European Neurogastroenterology and Motility Societies Neurogastroenterol Motil 2011 23 8 23 10.1111/j.1365-2982.2010.01612.x 21138500

[B3] Szarka LA Camilleri M Methods for measurement of gastric motility Am J Physiol 2009 296 G461 G475 10.1152/ajpgi.90467.2008 19147807

[B4] Lin HC Prather C Fisher RS Measurement of gastrointestinal transit Dig Dis Sci 2005 50 989 1004 15986844 10.1007/s10620-005-2694-6

[B5] Enck P Wienbeck M Repeated noninvasive measurement of gastrointestinal transit in rats Physiol Behav 1989 46 633 637 10.1016/0031-9384(89)90343-0 2602487

[B6] Baggio CH Freitas CS Rieck L Marques MC Gastroprotective effects of a crude extract of Baccharis illinita DC in rats Pharmacol Res 2003 47 93 98 10.1016/S1043-6618(02)00253-0 12526867

[B7] Miller MS Galligan JJ Burks TF Accurate measurement of intestinal transit in the rat J Pharmacol Meth 1981 6 211 217 10.1016/0160-5402(81)90110-8 7329070

[B8] Souza MA Souza MH Palheta RC Evaluation of gastrointestinal motility in awake rats: a learning exercise for undergraduate biomedical students Adv Physiol Educ 2009 33 343 348 10.1152/advan.90176.2008 19948686

[B9] Jain S Dani P Sharma RK Pharmacoscintigraphy: a blazing trail for the evaluation of new drugs and delivery systems Crit Rev Ther Drug Carr Syst 2009 26 373 426 10.1615/CritRevTherDrugCarrierSyst.v26.i4.20 20001891

[B10] Cora LA Americo MF Oliveira RB Biomagnetic methods: technologies applied to pharmaceutical research Pharm Res 2011 28 438 455 10.1007/s11095-010-0285-5 20949311

[B11] Maes BD Mys G Geypens BJ Gastric emptying flow curves separated from carbon-labeled octanoic acid breath test results Am J Physiol 1998 275 G169 G175 9655697 10.1152/ajpgi.1998.275.1.G169

[B12] Miranda JR Oliveira RB Sousa PL A novel biomagnetic method to study gastric antral contractions Phys Med Biol 1997 42 1791 1799 10.1088/0031-9155/42/9/010 9308084

[B13] Americo MF Oliveira RB Romeiro FG Scintigraphic validation of AC Biosusceptometry to study the gastric motor activity and the intragastric distribution of food in humans Neurogastroenterol Motil 2007 19 804 811 10.1111/j.1365-2982.2007.00960.x 17883432

[B14] Miranda JR Baffa O de Oliveira RB An AC biosusceptometer to study gastric emptying Med Phys 1992 19 445 448 10.1118/1.596832 1584144

[B15] Oliveira RB Baffa O Troncon LE Evaluation of a biomagnetic technique for measurement of orocaecal transit time Eur J Gastroenterol Hepatol 1996 8 491 495 8804879

[B16] Americo MF Marques RG Zandona EA Validation of ACB in vitro and in vivo as a biomagnetic method for measuring stomach contraction Neurogastroenterol Motil 2010 22 1340 1344, e1374 10.1111/j.1365-2982.2010.01582.x 20874731

[B17] Cora LA Romeiro FG Stelzer M AC biosusceptometry in the study of drug delivery Adv Drug Deliv Rev 2005 57 1223 1241 10.1016/j.addr.2005.01.026 15935871

[B18] Shimono N Takatori T Ueda M Chitosan dispersed system for colon-specific drug delivery Int J Pharm 2002 245 45 54 10.1016/S0378-5173(02)00344-7 12270241

[B19] Andreis U Americo MF Cora LA Gastric motility evaluated by electrogastrography and alternating current biosusceptometry in dogs Physiol Meas 2008 29 1023 1031 10.1088/0967-3334/29/9/002 18698113

[B20] Podczeck F Newton JM Yuen KH The description of the gastrointestinal transit of pellets assessed by gamma scintigraphy using statistical moments Pharm Res 1995 12 376 379 10.1023/A:1016200501563 7617524

[B21] Bratten J Jones MP New directions in the assessment of gastric function: clinical applications of physiologic measurements Dig Dis 2006 24 252 259 10.1159/000092878 16849852

[B22] Reynell PC Spray GH The simultaneous measurement of absorption and transit in the gastro-intestinal tract of the rat J Physiol 1956 131 452 462 13320346 10.1113/jphysiol.1956.sp005474 PMC1363441

[B23] Marcus CS Lengemann FW Use of radioyttrium to study food movement in the small intestine of the rat J Nutr 1962 76 179 182 14469700 10.1093/jn/76.2.179

[B24] Lorenzo CD Youssef NN Diagnosis and management of intestinal motility disorders Semin Pediatr Surg 2010 19 50 58 10.1053/j.sempedsurg.2009.11.006 20123274

[B25] Wald A Van Thiel DH Hoechstetter L Effect of pregnancy on gastrointestinal transit Dig Dis Sci 1982 27 1015 1018 10.1007/BF01391748 7140485

[B26] Forgacs I Patel V Diabetes and the gastrointestinal tract Medicine 2011 39 288 292 10.1016/j.mpmed.2011.02.007

[B27] Emmanuel A Raeburn A Small intestine and colon motility Medicine 2011 39 218 223 10.1016/j.mpmed.2011.01.002

[B28] Meunier LD Selection, acclimation, training, and preparation of dogs for the research setting ILAR J 2006 47 326 347 16963813 10.1093/ilar.47.4.326

[B29] Robinson V Less is more: reducing the reliance on animal models for nausea and vomiting research Br J Pharmacol 2009 157 863 864 10.1111/j.1476-5381.2009.00280.x 19624682 PMC2737645

[B30] Holmes AM Rudd JA Tattersall FD Opportunities for the replacement of animals in the study of nausea and vomiting Br J Pharmacol 2009 157 865 880 10.1111/j.1476-5381.2009.00176.x 19371333 PMC2737646

